# Super resolution microscopy is poised to reveal new insights into the formation and maturation of dendritic spines

**DOI:** 10.12688/f1000research.8649.1

**Published:** 2016-06-22

**Authors:** Cristina M. Robinson, Mikin R. Patel, Donna J. Webb

**Affiliations:** 1 Department of Biological Sciences, Vanderbilt University, Nashville, Tennessee, USA; 2 Vanderbilt Kennedy Center for Research on Human Development, Vanderbilt University, Nashville, Tennessee, USA; 3 Department of Cancer Biology, Vanderbilt University, Nashville, Tennessee, USA

**Keywords:** dendritic spines and synapses, neurological disorders, super resolution microscopy

## Abstract

Dendritic spines and synapses are critical for neuronal communication, and they are perturbed in many neurological disorders; however, the study of these structures in living cells has been hindered by their small size. Super resolution microscopy, unlike conventional light microscopy, is diffraction unlimited and thus is well suited for imaging small structures, such as dendritic spines and synapses. Super resolution microscopy has already revealed important new information about spine and synapse morphology, actin remodeling, and nanodomain composition in both healthy cells and diseased states. In this review, we highlight the advancements in probes that make super resolution more amenable to live-cell imaging of spines and synapses. We also discuss recent data obtained by super resolution microscopy that has advanced our knowledge of dendritic spine and synapse structure, organization, and dynamics in both healthy and diseased contexts. Finally, we propose a series of critical questions for understanding spine and synapse formation and maturation that super resolution microscopy is poised to answer.

## Introduction

Dendritic spines are actin-rich protrusions on neurons that are critical for neurotransmission, as they are sites for the majority of excitatory postsynapses ^1– 3^. Abnormal spines are found in a wide range of neuropsychiatric, neurodegenerative, and neurodevelopmental disorders ^4– 6^, further highlighting the importance of these structures in cognition. Spines typically consist of a thin neck and a bulbous head, which is 0.5 to 1 μm in diameter. Therefore, analyzing spine and synapse organization in detail was previously difficult owing to their small sizes, which are near the diffraction limit for conventional light microscopy ^7, 8^. The advent of super resolution imaging has revolutionized the study of spines and synapses. Whereas conventional light microscopy has an effective limit of resolution at ~200 nm due to the diffraction of light, super resolution fluorescence microscopy can bypass this limit, increasing the resolving power to tens of nanometers. In terms of resolving power, super resolution microscopy is limited by the brightness and photostability of the probes used ^9, 10^; the principles underlying super resolution microscopy have been discussed in detail in previous reviews ^7, 9, 11^. This enhanced resolving power enables more detailed examination of protein mobility in living cells. The live-cell application of super resolution microscopy is what currently sets it apart from electron microscopy, which can achieve a somewhat higher resolution (picometer) but is not compatible with live-cell imaging and requires stringent fixation conditions ^9^. Because super resolution microscopy is compatible with live-cell imaging, dynamic changes in spine and synapse morphology can be readily observed ^12– 14^. Particularly exciting is the possibility of imaging the very early stages of spine formation and subsequent maturation, which has not been possible to study with conventional light microscopy. Additionally, the enhanced resolving power of super resolution microscopy permits a more precise analysis of protein localization and the organization of protein nanodomains within individual spines and synapses ^15, 16^. This type of microscopy will be critical for detailing the organization and dynamics of the hundreds of proteins that are packed together in submicron structures, such as dendritic spines. Consequently, super resolution microscopy will enhance our knowledge of dendritic spine and synapse architecture to possibly reveal nanoscale abnormalities in diseased states and lend further insight into the mechanisms underlying neurodevelopmental disorders.

## Super resolution probes

New probes created in the last few years, as discussed below, have made super resolution microscopy even more conducive to visualizing neurons, especially fine neuronal structures (i.e. dendritic spines), because these probes are optimized for live-cell imaging. Super resolution studies are primarily performed using small molecule fluorophores and photoactivatable and photoswitchable fluorescent proteins ^17– 19^. Although small molecule fluorophores are less bulky, brighter, and more photostable compared to fluorescent protein tags, they can fail to bind to their intended targets and/or bind to undesired targets. However, by fusing a protein of interest directly to a fluorescent tag (i.e. green fluorescent protein [GFP]), this limitation can be overcome, but these fluorescently tagged proteins tend to be bulky and display weaker photostability and brightness than small molecule fluorophores. Over the past few years, researchers have focused on developing probes for super resolution microscopy that overcome the limitations of traditional fluorescent proteins and synthetic fluorophores. For example, quantum dots (QDs) ^20^, which are semiconductor nanoparticles, and nanobodies ^21^, which are composed of the smallest fragment of an antibody that will still bind to antigens, have also been used to label proteins for super resolution microscopy. The advantage of QDs is that they are highly photostable and therefore amenable to live-cell, single-molecule fluorescence microscopy. The major shortcoming for using QDs to visualize small neuronal structures is their size. QDs have an average diameter of 15–35 nm ^22^, making them difficult to utilize in spatially confined areas such as the synaptic cleft ^23^. To address this problem, Cai *et al*. developed small QDs (sQDs), which are about 7 nm in size and can easily label proteins in neuronal synapses ^22^. As a proof of concept, Wang *et al.* used sQDs conjugated to a GFP nanobody to label the exogenously expressed α-amino-3-hydroxy-5-methyl-4-isoxazole propionic acid receptor (AMPAR) subunit GluR2, which is fused to pH-sensitive GFP (pHluorin), to track the lateral diffusion of AMPARs in synapses ^24^. As an alternative approach to traditional fluorescent proteins, Viswanathan *et al.* created “spaghetti monster” fluorescent proteins (smFPs) ^25^ that contain multiple copies of commonly used tags such as Myc, FLAG, or HA on their surface. These tags create additional antibody binding sites on smFPs, which leads to high antibody labeling density, making these probes brighter than traditional fluorescence proteins or individual antibodies and nanobodies. Probe brightness is a critical determinant of spatial resolution in single-molecule super resolution microscopy. To test the ability of smFPs to reveal submicron structures, smFPs were expressed as a filler to visualize a major class of spines in CA3 neurons, called “thorny excrescence” spines. These spines have small protrusions from their spine neck, which are difficult to label with conventional fluorescent proteins and dyes. Even at low expression levels, smFPs labeled these protrusions significantly better than enhanced GFP (EGFP) or lucifer yellow ^25^. Furthermore, the epitope tags on smFPs allow for strong labeling of proteins for which suitable, specific antibodies and nanobodies are not available. Finally, smFPs are also especially attractive for investigating the early stages of spine and synapse formation because their brightness makes them well suited to imaging proteins that are present at low levels.

Actin is the main cytoskeletal element in dendritic spines and underlies spine morphology and plasticity. Despite its importance, until recently, super resolution live-cell imaging of actin remodeling in spines was limited to the use of low-affinity actin probes, such as ABP-tdEosFP ^26^ or exogenous expression of actin fused to a fluorescent protein ^19^. To address this, Lukinavičius *et al.* developed probes for live-cell imaging of actin and tubulin using a silicon-rhodamine derivative conjugated to ligands that bind to these cytoskeletal elements ^27^. The high specificity, enhanced fluorescence, and low phototoxicity of these probes make them invaluable for super resolution imaging of cytoskeletal remodeling in dendritic spines.

Collectively, the creation of these new probes has made super resolution microscopy even more amenable to studying small structures in neurons, such as dendritic spines and synapses, with unprecedented detail compared to conventional light microscopy. Although these probes overcome some of the weaknesses of older probes, newer probes are still needed that have all the characteristics of an ideal probe for imaging dendritic spines, including high specificity, brightness, and photostability, as well as small size.

## Novel insights from super resolution microscopy

### A new view of the actin cytoskeleton in spines

Although actin remodeling, which is critical for dendritic spine morphology and structure, has been studied in spines using conventional light microscopy, super resolution microscopy is providing important new information regarding the actin cytoskeleton in spines. Tatavarty *et al.* showed that the incorporation of individual actin monomers into actin filaments is more complex and heterogeneous than originally demonstrated with confocal microscopy ^19^. In spines, single actin filaments were found to undergo retrograde flow, while other individual filaments displayed anterograde flow, random motion, or no net movement. This heterogeneity of actin polymerization in spines was confirmed by Frost *et al.* ^28^. Furthermore, they demonstrated that certain subdomains in spines, such as the postsynaptic density (PSD) and spine neck, exhibit enhanced actin polymerization ^28^. Super resolution microscopy also revealed that approximately 70% of spines that appear globular or cup-shaped by confocal microscopy display finger-like membrane extensions, which were driven by filamentous-actin (F-actin) dynamics ^12^. Intriguingly, the nucleation of these extensions may not occur at the tip of the extension, as previously thought for other membrane protrusions ^29^. Instead, Abi1 and Nap1, which are components of the actin-nucleating WAVE complex, localized at a single, central domain at the PSD ^12^, suggesting that the extensions are initially nucleated at the PSD. This raises the interesting possibility that these extensions play a role in spine maturation by sensing changes in the local environment and relaying this information back to the PSD. In addition, when the actin cytoskeleton was disrupted by treatment with cytochalasin D, synaptopodin, which localizes to the spine neck ^30^, no longer regulated diffusion of the metabotropic glutamate receptor 5 (mGluR5) ^31^. These results suggest that components of the actin cytoskeleton are critical for the synaptopodin-mediated effect on diffusion. Super resolution microscopy has already provided new insight into actin remodeling in stable spines, and it has the potential to reveal critical new information about actin structure and function in dendritic spine and synapse assembly and maturation.

### The importance of nanodomains

Super resolution microscopy has been used to visualize protein nanodomains within both the PSD and other areas of the spine. The importance of these nanodomains in neuronal function is also beginning to become evident (reviewed by MacGillavry and Hoogenraad ^32^). Different individual nanodomains of the same protein display different life times and changes in morphology over time. For example, while 40% of AMPAR nanodomains do not remain stable for longer than 5 minutes, 20% persist for at least 1 hour ^15^. Additionally, the morphology of PSD95 nanodomains has also been found to change with time ^33^. Intriguingly, when neurons were treated with tetrodotoxin, which blocks sodium channels to prevent neural signaling, the area of the PSD was increased ^33^, suggesting that the nanodomain composition within the PSD changes in response to neural activity. Less clear, though, is how protein nanodomains are established during neuronal development and how they change over time in response to synaptic plasticity. A few studies have analyzed the changes in nanodomains in response to glutamate receptor activation or chemical long-term potentiation (LTP) ^34, 35^. For example, Lu *et al*. examined the mobility of calcium/calmodulin-dependent kinase II (CamKII), a protein consisting of α and β subunits which is necessary for inducing LTP and plays a role in trafficking AMPARs into synapses ^36^. CamKIIα was found to exist in three kinetic populations: slow, intermediate, and fast ^34^. Each population was associated with different binding partners, where the fast population was found to be the CamKIIα subunit alone, the intermediate population consisted of the α subunit bound to the β subunit and F-actin, and the slow population was thought to be CamKII bound to immobile substrates. Interestingly, stimulation of N-methyl-D-aspartate receptors (NMDARs) by glutamate and glycine significantly decreased CamKII mobility both at the PSD and elsewhere in spines, suggesting that CamKII is important for not only modulating AMPAR density in synapses but functions elsewhere in spines. Moreover, nanodomains of ankyrin-G, an adaptor protein that is a risk factor for schizophrenia, autism, and bipolar disorder ^37– 39^, accumulate in spines in response to chemical LTP ^35^. Knockdown of ankyrin-G prevents increases in spine head enlargement, a correlate for spine maturity and synapse size ^40, 41^, following chemical LTP stimulation ^35^. Intriguingly, there was no difference in spine head size between spines that contained ankyrin-G in the spine neck prior to LTP and those which contained ankyrin-G in the spine neck after LTP. This suggests that the presence of ankyrin-G in the spine neck is a marker for spines that have already fully matured. Interestingly, another protein involved in synapse organization, synaptic cell adhesion molecule 1 (SynCAM 1), displayed an increase in nanodomain size in response to long-term depression ^42^. Collectively, these data indicate that changes to nanodomain composition and characteristics are key for altering synaptic strength and suggest that changes to nanodomain composition occur during different stages of spine development.

To date, super resolution microscopy has not been used to examine the formation of protein nanodomains in developing spines. However, data obtained from stable spines could be applicable to forming spines as well. For instance, Hruska *et al.* used super resolution microscopy to show that the neuronal adhesion protein ephrin B3 regulates the localization of PSD95 to stable synapses and that ephrin B3, but not other, related ephrins, is critical for stabilizing PSD95 nanodomains in spines ^43^. Interestingly, neuronal activity stimulated the phosphorylation of ephrin B3 at serine 332 (S332), which decreased ephrin B3 localization to synapses and impaired its interaction with PSD95 ^43^. Thus, ephrin B3, when not phosphorylated at S332, may be critical for recruiting PSD95 to sites where new synapses are forming ^43^. Indeed, knockdown of either PSD95 or ephrin B3 decreases spine density ^44, 45^; however, whether this effect is due to decreased spine maintenance or formation is not currently known.

### Abnormal spines in diseased states

Using conventional light microscopy, alterations in dendritic spine size, number, and morphology have been found in neurological disorders such as Alzheimer’s disease ^46, 47^, schizophrenia ^48, 49^, and Fragile X syndrome (FXS) ^50, 51^. While confocal microscopy is limited to 200 nm resolution, super resolution microscopy can potentially provide detailed insights into the structural changes and nanodomain composition of dendritic spines seen in these disorders. Presently, a few studies have examined the structural changes to dendritic spines in neurological and neurodegenerative disorders. Using super resolution microscopy, Šišková *et al.* observed that dendritic branching, dendritic length, and dendritic surface area in CA1 pyramidal neurons from an Alzheimer’s mouse model were significantly reduced compared to those from wild-type (WT) mice ^52^. Classically, confocal microscopy has shown that FXS is associated with an increase in long, thin, filopodia-like, immature spines. However, Wijetunge *et al.* found unexpected results when examining changes in spine density and morphology between WT mice and FXS model mice (Fragile X mental retardation protein knockout mice) ^53^. The spine densities in hippocampal and cortical brain regions from FXS mice were comparable to those observed with WT mice when imaged via super resolution microscopy. However, subtle changes in fine morphological structures such as neck length, neck width, and head size were observed during different developmental stages. Moreover, Barnes *et al*. also showed that animals from another mouse model for intellectual disability (SynGAP ^+/-^) display no significant change in spine density but instead show increased spine neck length and decreased neck width, leading to increased compartmentalization, compared to WT mice ^54^. Intriguingly, they demonstrated that common physiological pathways are disrupted in the SynGAP heterozygous model and the FXS model, leading to similar morphological changes in dendritic spines in both. Together, these findings suggest that abnormalities observed in dendritic spine morphology and density in diseased states are both developmental stage and brain region specific and that these changes are the result of disruptions in pathways shared by multiple diseases. Further research is needed to better understand the functional implications of structural abnormalities in dendritic spines in these and other neurological disorders.

## Conclusions and future directions

Although the proper development of dendritic spines and synapses is critical for normal cognitive function, their small size has limited the acquisition of detailed images of their nanoscopic substructures via conventional light microscopy. Super resolution microscopy overcomes the diffraction barrier, which allows for the imaging of these structures. While electron microscopy can achieve even higher resolution, it is limited because it cannot currently be performed in living cells. In contrast, super resolution microscopy is amenable to live-cell imaging. Moreover, super resolution microscopy will be critical for visualizing interactions between actin and actin-binding proteins during the early stages of dendritic spine formation and their subsequent maturation. The recent development of probes that are smaller, brighter, more specific, and more conducive to live-cell imaging are turning super resolution microscopy into a vital new tool to better understand dendritic spine morphology, organization, function, and plasticity. Indeed, super resolution microscopy has already revealed fascinating and important new information about both the gross anatomical structure of spines and the protein nanodomain composition as well as actin remodeling within them in both healthy tissue and in diseased states. Super resolution microscopy will be invaluable to many applications in neuroscience, but it specifically offers the potential to examine spine and synapse development at a level of detail in living cells which was previously not possible but is necessary to understand the underlying mechanisms that regulate this process.

Super resolution microscopy can now be used to address a number of intriguing questions about the development of dendritic spines. For example, when are synaptic nanodomains established during spine formation, and how do they affect filopodia and spine morphology? Do all synaptic proteins enter a forming spine simultaneously, or are they recruited sequentially? Which protein nanodomains assemble independently, and which domains require synaptic scaffolding proteins to assemble appropriate nanodomains? How does nanodomain composition correlate with overall spine morphology? For instance, are the properties of nanodomains in a developing filopodium the same as what is seen in mature synapses, or are there immature stages, where nanodomains show different properties in immature synapses? The answers to these and other interesting questions would lend insight into novel functions for synaptic proteins. It will be critical to not only assess the normal development of dendritic spines but also evaluate how spine formation and maturation are perturbed in neurological disorders, such as Alzheimer’s disease, schizophrenia, and FXS. Super resolution imaging has the potential to reveal the mechanisms that underlie these abnormalities and allow for the generation of new treatments for these disorders.

## References

[1] Alvarez VA Sabatini BL: Anatomical and physiological plasticity of dendritic spines. *Annu Rev Neurosci.* 2007;30:79–97. 10.1146/annurev.neuro.30.051606.094222 17280523

[2] Ebrahimi S Okabe S: Structural dynamics of dendritic spines: molecular composition, geometry and functional regulation. *Biochim Biophys Acta.* 2014;1838(10):2391–8. 10.1016/j.bbamem.2014.06.002 24915021

[3] Jan YN Jan LY: Dendrites. *Genes Dev.* 2001;15(20):2627–41. 10.1101/gad.916501 11641269

[4] Fiala JC Spacek J Harris KM: Dendritic spine pathology: cause or consequence of neurological disorders? *Brain Res Brain Res Rev.* 2002;39(1):29–54. 10.1016/S0165-0173(02)00158-3 12086707

[5] Kulkarni VA Firestein BL: The dendritic tree and brain disorders. *Mol Cell Neurosci.* 2012;50(1):10–20. 10.1016/j.mcn.2012.03.005 22465229

[6] Penzes P Cahill ME Jones KA: Dendritic spine pathology in neuropsychiatric disorders. *Nat Neurosci.* 2011;14(3):285–93. 10.1038/nn.2741 21346746 PMC3530413

[7] Castro JB Gould TJ: Neuro at the Nanoscale: Diffraction-Unlimited Imaging with STED Nanoscopy. *J Histochem Cytochem.* 2015;63(12):897–907. 10.1369/0022155415610169 26392517 PMC4823800

[8] Sheng M Kim E: The postsynaptic organization of synapses. *Cold Spring Harb Perspect Biol.* 2011;3(12): pii: a005678. 10.1101/cshperspect.a005678 22046028 PMC3225953

[9] Rutherford MA: Resolving the structure of inner ear ribbon synapses with STED microscopy. *Synapse.* 2015;69(5):242–55. 10.1002/syn.21812 25682928

[10] Fernández-Suárez M Ting AY: Fluorescent probes for super-resolution imaging in living cells. *Nat Rev Mol Cell Biol.* 2008;9(12):929–43. 10.1038/nrm2531 19002208

[11] Huang B Babcock H Zhuang X: Breaking the diffraction barrier: super-resolution imaging of cells. *Cell.* 2010;143(7):1047–58. 10.1016/j.cell.2010.12.002 21168201 PMC3272504

[12] Chazeau A Mehidi A Nair D: Nanoscale segregation of actin nucleation and elongation factors determines dendritic spine protrusion. *EMBO J.* 2014;33(23):2745–64. 10.15252/embj.201488837 25293574 PMC4282554

[13] Ding JB Takasaki KT Sabatini BL: Supraresolution imaging in brain slices using stimulated-emission depletion two-photon laser scanning microscopy. *Neuron.* 2009;63(4):429–37. 10.1016/j.neuron.2009.07.011 19709626 PMC2756148

[14] Nägerl UV Willig KI Hein B: Live-cell imaging of dendritic spines by STED microscopy. *Proc Natl Acad Sci U S A.* 2008;105(48):18982–7. 10.1073/pnas.0810028105 19028874 PMC2585941

[15] Nair D Hosy E Petersen JD: Super-resolution imaging reveals that AMPA receptors inside synapses are dynamically organized in nanodomains regulated by PSD95. *J Neurosci.* 2013;33(32):13204–24. 10.1523/JNEUROSCI.2381-12.2013 23926273 PMC6619720

[16] Westin L Reuss M Lindskog M: Nanoscopic spine localization of Norbin, an mGluR5 accessory protein. *BMC Neurosci.* 2014;15:45. 10.1186/1471-2202-15-45 24670218 PMC3976536

[17] Blom H Rönnlund D Scott L: Nearest neighbor analysis of dopamine D1 receptors and Na ^+^-K ^+^-ATPases in dendritic spines dissected by STED microscopy. *Microsc Res Tech.* 2012;75(2):220–8. 10.1002/jemt.21046 21809413

[18] Kaminski Schierle GS van de Linde S Erdelyi M: *In situ* measurements of the formation and morphology of intracellular β-amyloid fibrils by super-resolution fluorescence imaging. *J Am Chem Soc.* 2011;133(33):12902–5. 10.1021/ja201651w 21793568

[19] Tatavarty V Kim EJ Rodionov V: Investigating sub-spine actin dynamics in rat hippocampal neurons with super-resolution optical imaging. *PLoS One.* 2009;4(11):e7724. 10.1371/journal.pone.0007724 19898630 PMC2771285

[20] Uematsu M Adachi E Nakamura A: Atomic identification of fluorescent Q-dots on tau-positive fibrils in 3D-reconstructed pick bodies. *Am J Pathol.* 2012;180(4):1394–7. 10.1016/j.ajpath.2011.12.029 22322305

[21] Mikhaylova M Cloin BM Finan K: Resolving bundled microtubules using anti-tubulin nanobodies. *Nat Commun.* 2015;6:7933. 10.1038/ncomms8933 26260773 PMC4918323

[22] Cai E Ge P Lee SH: Stable small quantum dots for synaptic receptor tracking on live neurons. *Angew Chem Int Ed Engl.* 2014;53(46):12484–8. 10.1002/anie.201405735 25255882 PMC4240739

[23] Groc L Lafourcade M Heine M: Surface trafficking of neurotransmitter receptor: comparison between single-molecule/quantum dot strategies. *J Neurosci.* 2007;27(46):12433–7. 10.1523/JNEUROSCI.3349-07.2007 18003820 PMC6673310

[24] Wang Y Cai E Rosenkranz T: Small quantum dots conjugated to nanobodies as immunofluorescence probes for nanometric microscopy. *Bioconjug Chem.* 2014;25(12):2205–11. 10.1021/bc5004179 25397889 PMC4275168

[25] Viswanathan S Williams ME Bloss EB: High-performance probes for light and electron microscopy. *Nat Methods.* 2015;12(6):568–76. 10.1038/nmeth.3365 25915120 PMC4573404

[26] Izeddin I Specht CG Lelek M: Super-resolution dynamic imaging of dendritic spines using a low-affinity photoconvertible actin probe. *PLoS One.* 2011;6(1):e15611. 10.1371/journal.pone.0015611 21264214 PMC3022016

[27] Lukinavičius G Blaukopf C Pershagen E: SiR-Hoechst is a far-red DNA stain for live-cell nanoscopy. *Nat Commun.* 2015;6: 8497. 10.1038/ncomms9497 26423723 PMC4600740

[28] Frost NA Shroff H Kong H: Single-molecule discrimination of discrete perisynaptic and distributed sites of actin filament assembly within dendritic spines. *Neuron.* 2010;67(1):86–99. 10.1016/j.neuron.2010.05.026 20624594 PMC2904347

[29] Hotulainen P Hoogenraad CC: Actin in dendritic spines: connecting dynamics to function. *J Cell Biol.* 2010;189(4):619–29. 10.1083/jcb.201003008 20457765 PMC2872912

[30] Deller T Merten T Roth SU: Actin-associated protein synaptopodin in the rat hippocampal formation: localization in the spine neck and close association with the spine apparatus of principal neurons. *J Comp Neurol.* 2000;418(2):164–81. 10.1002/(SICI)1096-9861(20000306)418:2<164::AID-CNE4>3.0.CO;2-0 10701442

[31] Wang L Dumoulin A Renner M: The Role of Synaptopodin in Membrane Protein Diffusion in the Dendritic Spine Neck. *PLoS One.* 2016;11(2):e0148310. 10.1371/journal.pone.0148310 26840625 PMC4739495

[32] MacGillavry HD Hoogenraad CC: The internal architecture of dendritic spines revealed by super-resolution imaging: What did we learn so far? *Exp Cell Res.* 2015;335(2):180–6. 10.1016/j.yexcr.2015.02.024 25746722

[33] MacGillavry HD Song Y Raghavachari S: Nanoscale scaffolding domains within the postsynaptic density concentrate synaptic AMPA receptors. *Neuron.* 2013;78(4):615–22. 10.1016/j.neuron.2013.03.009 23719161 PMC3668352

[34] Lu HE MacGillavry HD Frost NA: Multiple spatial and kinetic subpopulations of CaMKII in spines and dendrites as resolved by single-molecule tracking PALM. *J Neurosci.* 2014;34(22):7600–10. 10.1523/JNEUROSCI.4364-13.2014 24872564 PMC4035521

[35] Smith KR Kopeikina KJ Fawcett-Patel JM: Psychiatric risk factor *ANK3*/ankyrin-G nanodomains regulate the structure and function of glutamatergic synapses. *Neuron.* 2014;84(2):399–415. 10.1016/j.neuron.2014.10.010 25374361 PMC4223651

[36] Lisman J Schulman H Cline H: The molecular basis of CaMKII function in synaptic and behavioural memory. *Nat Rev Neurosci.* 2002;3(3):175–90. 10.1038/nrn753 11994750

[37] Cruz DA Weaver CL Lovallo EM: Selective alterations in postsynaptic markers of chandelier cell inputs to cortical pyramidal neurons in subjects with schizophrenia. *Neuropsychopharmacology.* 2009;34(9):2112–24. 10.1038/npp.2009.36 19322171 PMC2721024

[38] Iqbal Z Vandeweyer G van der Voet M: Homozygous and heterozygous disruptions of *ANK3:* at the crossroads of neurodevelopmental and psychiatric disorders. *Hum Mol Genet.* 2013;22(10):1960–70. 10.1093/hmg/ddt043 23390136

[39] Schulze TG Detera-Wadleigh SD Akula N: Two variants in *Ankyrin 3 (ANK3)* are independent genetic risk factors for bipolar disorder. *Mol Psychiatry.* 2009;14(5):487–91. 10.1038/mp.2008.134 19088739 PMC2793269

[40] Harris KM Jensen FE Tsao B: Three-dimensional structure of dendritic spines and synapses in rat hippocampus (CA1) at postnatal day 15 and adult ages: implications for the maturation of synaptic physiology and long-term potentiation. *J Neurosci.* 1992;12(7):2685–705. 1613552 10.1523/JNEUROSCI.12-07-02685.1992 PMC6575840

[41] Schikorski T Stevens CF: Quantitative fine-structural analysis of olfactory cortical synapses. *Proc Natl Acad Sci U S A.* 1999;96(7):4107–12. 10.1073/pnas.96.7.4107 10097171 PMC22428

[42] Perez de Arce K Schrod N Metzbower SW: Topographic Mapping of the Synaptic Cleft into Adhesive Nanodomains. *Neuron.* 2015;88(6):1165–72. 10.1016/j.neuron.2015.11.011 26687224 PMC4687029

[43] Hruska M Henderson NT Xia NL: Anchoring and synaptic stability of PSD-95 is driven by ephrin-B3. *Nat Neurosci.* 2015;18(11):1594–605. 10.1038/nn.4140 26479588 PMC5396457

[44] Ehrlich I Klein M Rumpel S: PSD-95 is required for activity-driven synapse stabilization. *Proc Natl Acad Sci U S A.* 2007;104(10):4176–81. 10.1073/pnas.0609307104 17360496 PMC1820728

[45] McClelland AC Hruska M Coenen AJ: Trans-synaptic EphB2-ephrin-B3 interaction regulates excitatory synapse density by inhibition of postsynaptic MAPK signaling. *Proc Natl Acad Sci U S A.* 2010;107(19):8830–5. 10.1073/pnas.0910644107 20410461 PMC2889310

[46] Knafo S Alonso-Nanclares L Gonzalez-Soriano J: Widespread changes in dendritic spines in a model of Alzheimer's disease. *Cereb Cortex.* 2009;19(3):586–92. 10.1093/cercor/bhn111 18632740

[47] Ji Y Gong Y Gan W: Apolipoprotein E isoform-specific regulation of dendritic spine morphology in apolipoprotein E transgenic mice and Alzheimer's disease patients. *Neuroscience.* 2003;122(2):305–15. 10.1016/j.neuroscience.2003.08.007 14614898

[48] Pathania M Davenport EC Muir J: The autism and schizophrenia associated gene CYFIP1 is critical for the maintenance of dendritic complexity and the stabilization of mature spines. *Transl Psychiatry.* 2014;4:e374. 10.1038/tp.2014.16 24667445 PMC3966042

[49] Glantz LA Lewis DA: Decreased dendritic spine density on prefrontal cortical pyramidal neurons in schizophrenia. *Arch Gen Psychiatry.* 2000;57(1):65–73. 10.1001/archpsyc.57.1.65 10632234

[50] Comery TA Harris JB Willems PJ: Abnormal dendritic spines in fragile X knockout mice: maturation and pruning deficits. *Proc Natl Acad Sci USA.* 1997;94(10):5401–4. 10.1073/pnas.94.10.5401 9144249 PMC24690

[51] Irwin SA Patel B Idupulapati M: Abnormal dendritic spine characteristics in the temporal and visual cortices of patients with fragile-X syndrome: a quantitative examination. *Am J Med Genet.* 2001;98(2):161–7. 10.1002/1096-8628(20010115)98:2<161::AID-AJMG1025>3.0.CO;2-B 11223852

[52] Šišková Z Justus D Kaneko H: Dendritic structural degeneration is functionally linked to cellular hyperexcitability in a mouse model of Alzheimer's disease. *Neuron.* 2014;84(5):1023–33. 10.1016/j.neuron.2014.10.024 25456500

[53] Wijetunge LS Angibaud J Frick A: Stimulated emission depletion (STED) microscopy reveals nanoscale defects in the developmental trajectory of dendritic spine morphogenesis in a mouse model of fragile X syndrome. *J Neurosci.* 2014;34(18):6405–12. 10.1523/JNEUROSCI.5302-13.2014 24790210 PMC4004821

[54] Barnes SA Wijetunge LS Jackson AD: Convergence of Hippocampal Pathophysiology in *Syngap ^+/-^* and *Fmr1 ^-/y^* Mice. *J Neurosci.* 2015;35(45):15073–81. 10.1523/JNEUROSCI.1087-15.2015 26558778 PMC4642239

